# Deciphering mouse uterine receptivity for embryo implantation at single‐cell resolution

**DOI:** 10.1111/cpr.13128

**Published:** 2021-09-23

**Authors:** Yi Yang, Qiu‐Yang Zhu, Ji‐Long Liu

**Affiliations:** ^1^ College of Veterinary Medicine Gansu Agricultural University Lanzhou China; ^2^ Guangdong Laboratory for Lingnan Modern Agriculture College of Veterinary Medicine South China Agricultural University Guangzhou China

## Abstract

**Objectives:**

Mice are widely used as an animal model for studying human uterine receptivity for embryo implantation. Although transcriptional changes related to mouse uterine receptivity have been determined by using bulk RNA‐seq, the data are of limited value because the uterus is a complex organ consisting of many cell types. Here, we aimed to decipher mouse uterine receptivity for embryo implantation at single‐cell resolution.

**Materials and methods:**

Single‐cell RNA sequencing was performed for the pre‐receptive and the receptive mouse uterus. Gene expression profiles in luminal epithelium and glandular epithelium were validated by comparing against a published laser capture microdissection (LCM)‐coupled microarray dataset.

**Results:**

We revealed 19 distinct cell clusters, including 3 stromal cell clusters, 2 epithelial cell clusters, 1 smooth muscle cell cluster, 4 endothelial cell clusters and 8 immune cell clusters. We identified global gene expression changes associated with uterine receptivity in each cell type. Additionally, we predicted signalling interactions for distinct cell types to understand the crosstalk between the blastocyst and the receptive uterus.

**Conclusion:**

Our data provide a valuable resource for deciphering the molecular mechanism underlying uterine receptivity in mice.

## Graphical abstract

In this study, by using the state‐of‐the‐art single‐cell RNA‐seq approach, we resolved all cell types of the pre‐receptive and the receptive mouse uterus at single‐cell resolution. Consequently, we were able to identify global gene expression changes associated with uterine receptivity in each cell type. Additionally, we predicted signaling interactions for distinct cell types to understand the crosstalk between the blastocyst and the receptive uterus. Our data provide a valuable resource for deciphering the molecular mechanism underlying uterine receptivity in mice.

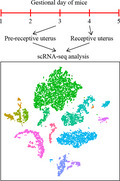

## 1 INTRODUCTION

Embryo implantation is a primary bottleneck step for human reproduction: the pregnancy rate per menstrual cycle is approximately 30%, mainly due to implantation failure.^1^, ^2^ Successful embryo implantation depends on an implantation competent embryo and a receptive uterus.^3^ It has been estimated that the embryonic factor contributes to two thirds of implantation failures, while the maternal factor, that is inadequate uterine receptivity, is responsible for the other one third.^4^ In assisted reproductive technologies, despite the high success rate of in vitro fertilization, the implantation rate remains very low.^5^ Therefore, it is imperative to understand the molecular mechanism involved in establishing uterine receptivity for embryo implantation.

Due to ethical restrictions and experimental difficulties, studies on human uterine receptivity are mostly limited to in vitro experiments.^6^, ^7^ In vivo probing of uterine receptivity heavily relies on mice.^3^ By using gene knockout mice, a number of genes have been proved to play key roles in uterine receptivity.^8^, ^9^, ^10^, ^11^, ^12^, ^13^ Previously, by using RNA‐seq, we determined global gene expression changes associated with uterine receptivity in mice.^14^ We revealed 541 differentially expressed genes with 316 genes being up‐regulated and 225 genes being down‐regulated in the receptive uterus compared to the pre‐receptive uterus, presenting a useful candidate gene list for further study of uterine receptivity in mice. However, a clear limitation is that the whole uterus was used for RNA‐seq analysis. The uterus is a complex structure consisting of three layers, endometrium, myometrium and perimetrium, with many cell types, including luminal and glandular epithelial cells, stromal cells, smooth muscle cells, endothelial cells and various immune cells. For a highly heterogeneous organ such as the uterus, the conventional bulk RNA‐seq approach is unable to accurately capture cell‐type‐specific gene expression changes.^15^

With advances in the single‐cell RNA‐seq techniques, it is now possible to analyse global gene expression profiles within a highly heterogeneous organ at single‐cell level.^16^ In this study, by using the state‐of‐the‐art single‐cell RNA‐seq approach, we resolved all cell types of the pre‐receptive and the receptive mouse uterus at single‐cell resolution. Consequently, we were able to identify global gene expression changes associated with uterine receptivity for each cell type. Additionally, we predicted signalling interactions for distinct cell types to understand the crosstalk between the blastocyst and the receptive uterus. Our study provides a valuable resource for deciphering the molecular mechanism underlying uterine receptivity in mice.

## 2 MATERIALS AND METHODS

### 2.1 *Sample* *collection*

Adult CD‐1 mice of the specific‐pathogen free (SPF) grade were used in this study. All mice were caged under light‐controlled conditions (14‐h/10‐h light/dark cycles) with free access to regular food and water. Female mice were mated with fertile males, and the mating was confirmed the next morning by the presence of a vaginal plug. The day of the vaginal plug was denoted as gestation day (GD) 1. The whole uterus was obtained at 0900 h on GD3 (pre‐receptive stage) and GD4 (receptive stage) respectively. Success of breeding and early embryo development was further confirmed by recovering morula from the oviduct on GD3. On GD4, one horn of the uterus was flushed with saline and early embryo development was further confirmed by recovering blastocysts from the uterus. The other intact uterine horn was used for sample collection. All animal procedures were approved by the Institutional Animal Care and Use Committee of South China Agricultural University.

### 2.2 *Haematoxylin* *and eosin staining*

Uterine tissues were fixed in 4% paraformaldehyde solution for 24 h. After paraffin processing, tissues were cut into 6‐μm sections and stained with haematoxylin and eosin.

### 2.3 Immunohistochemistry

Paraformaldehyde‐fixed paraffin‐embedded uterine samples were cut into 6 μm sections. Antigen retrieval was performed by heating the slides in 10 mM citrate buffer for 10 min. Endogenous peroxidase activity was removed by using 3% H_2_O_2_. After blocking with 10% horse serum in PBS, sections were incubated with anti‐FOXA2 (1:200 dilution, #ab108422, Abcam) or normal rabbit IgG in 10% horse serum overnight at 4°C. The signal was developed by the 3,3'‐diaminobenzidine (DAB)‐HRP reaction system. Sections were counterstained with haematoxylin. The positive signal was visualized as a dark brown colour.

### 2.4 *Bulk* *RNA*‐*seq analysis*

The total RNA from uterine tissues was extracted with the TRIzol reagent (Invitrogen). RNA‐seq libraries were generated by using the TruSeq RNA sample preparation kit (Illumina) and sequenced on a HiSeq 2500 system (Illumina). Raw data were trimmed by the fastp program^17^ with the parameters ‘‐w 4 ‐q 20 ‐u 50’ to obtain clean reads. Clean reads were mapped to mouse genome UCSC mm10 by using Hisat2 software v2.0.5^18^ with default parameters. HTSeq v0.13.5^19^ was subsequently employed to convert aligned short reads into read counts for each gene. Gene expression levels were normalized as transcripts per kilobase million (TPM) using an in‐house PERL script.

### 2.5 *Single*‐*cell* *dissociation of mouse uterus*

The uterine tissues from 3 mice for each group were pooled and minced with a blade. Tissues were then incubated in the dissociation buffer containing 2 mg/ml Collagenase II (#C6885, Sigma‐Aldrich), 10 mg/ml Dispase II (#354235, Corning) and 50,000 U/ml DNase I (#DN25, Sigma‐Aldrich) for up to 30 min at 37°C in a shaking incubator. The digestion progress was checked every 5 min with a microscope until single‐cell suspension was achieved. The single‐cell suspension was then passed through a 40‐μm cell strainer to remove undigested tissues. Cells were spun down at 250 g at 4°C for 4 min, and the pelleted cells were washed using centrifugation. In order to measure cell viability, cells were stained with AO/PI solution (#CS2‐0106, Nexcelom Bioscience) and counted using a Cellometer Auto 2000 instrument (#SD‐100, Nexcelom Bioscience). The single‐cell suspension was carried forward to single‐cell RNA‐seq only if the cell viability was >80% and the percentage of cell clumps was <10%.

### 2.6 *Single*‐*cell* *RNA*‐*seq library preparation and sequencing*

The final concentration of single‐cell suspension was adjusted to 1000 cells/μl, and a volume of 15 µl was loaded into one channel of the Chromium™ Single Cell B Chip (#1000073, 10x Genomics), aiming at recovering 8000–10,000 cells. The Chromium Single Cell 3’ Library & Gel Bead Kit v3 (#1000075, 10x Genomics) was used for single‐cell bar‐coding, cDNA synthesis and library preparation, following the manufacturer's instructions provided as the Single Cell 3’ Reagent Kits User Guide Version 3. Library sequencing was performed on a NovaSeq 6000 system (Illumina) configured with the paired‐end 150 bp protocol at a sequencing depth of approximately 400 million reads.

### 2.7 *Single*‐*cell* *RNA*‐*seq data analysis*

Raw data of bcl files from the NovaSeq 6000 system were converted to fastq files using the bcl2fastq2 tool v2.19.0.316 (Illumina). These fastq files were aligned to the mm10 mouse reference genome by using the CellRanger software v3.0.1 (10x Genomics). The resulting gene counts matrix was processed with the R package Seurat v3.1.3.^20^ Cells with fewer than 200 or greater than 6000 unique genes, as well as cells with greater than 25% of mitochondrial counts, were excluded. Meanwhile, genes expressed in fewer than 3 cells were removed. Following data filtering, the clean gene counts matrix was normalized and scaled by using NormalizeData and ScaleData respectively. The top 2000 highest variable genes were used for the principal component analysis (PCA), and the optimal number of PCA components was determined by the JackStraw procedure. Single cells were clustered by the graph‐based algorithm in PCA space and visualized using the t‐distributed stochastic neighbour embedding (tSNE) dimensional reduction technique. The cell‐type label for each cell cluster was manually assigned based on canonical cell markers. The FindAllMarkers function was used to identify novel marker genes for each cluster with a minimum of 20% of cells expressing the gene within the cluster and a minimum logFC threshold of 0.25. In order to find differential expressed genes in the same cell type between pre‐receptive uterus and receptive uterus, the FindMarkers function in Seurat was used with min.pct being set to 0.20 and min.logfc being set to 0.25.

### 2.8 *Gene* *ontology analysis*

Gene ontology (GO) analysis was performed as described previously.^21^ GO terms were grouped according to the biological process category in the Mouse Genome Informatics (MGI) GOslim database.^22^ To test for enrichment, a hypergeometric test was conducted and *p *= 0.05 was used as significance threshold to identify enriched GO terms.

### 2.9 *Gene* *network reconstruction*

Gene network reconstruction was performed by using the STRING online tool v11.0.^23^ The threshold score for gene‐gene interaction was set to 0.9. The Cytoscape software v2.8.1 was used to display the network.^24^ The Network Analyzer plugin for Cytoscape was used to compute degree distribution.^25^ Hub genes in the network were selected by using a defined degree cut‐off value of mean + 2 × SD.

### 2.10 *Cell*‐*cell* *communication analysis*

The single‐cell RNA‐seq data for mouse E3.5 blastocysts were downloaded from the GEO database (GSM4026212)^26^ and re‐analysed with the same pipeline as described above. E3.5 is equivalent to GD4. In order to analyse the cell‐cell communication between blastocysts and uterus, we merged E3.5 blastocyst data and GD4 uterus data into a single Seurat object, from which a meta file as well as a count file was generated. These 2 files were used as input for the CellphoneDB software v2.1.4 to infer cell‐cell communications based on ligand‐receptor interactions with default parameters.^27^ *p *< 0.05 were considered significant.

## 3 RESULTS

### 3.1 *Identification* *of cell types in mouse uterus*

**FIGURE 1:**
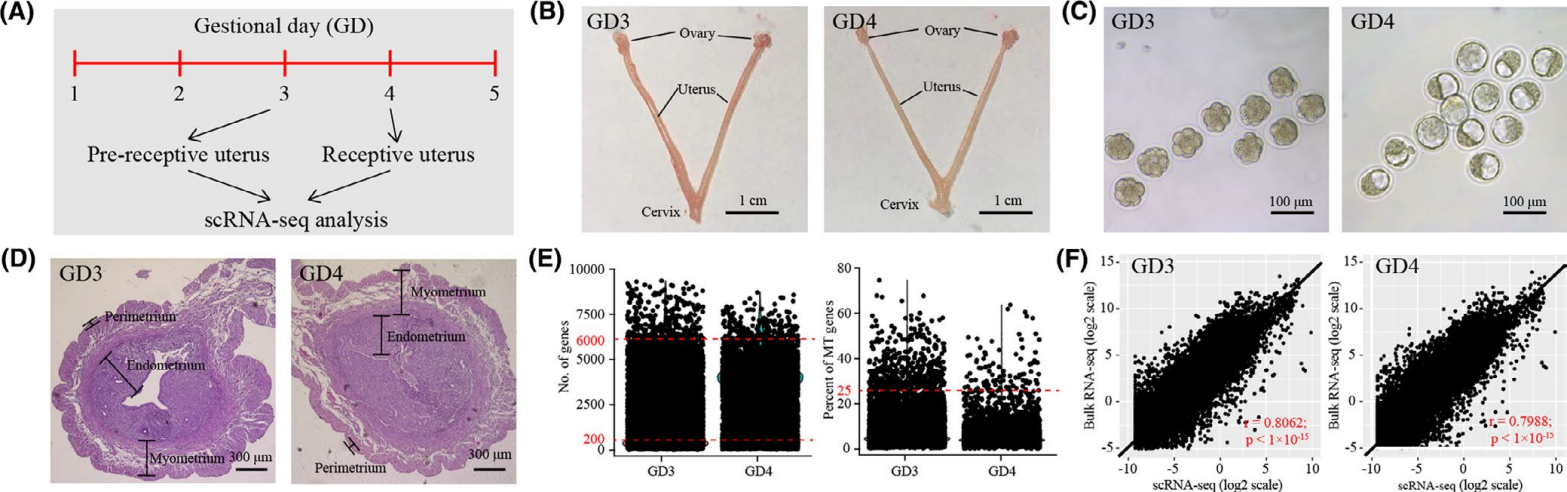
Single‐cell transcriptome analysis of uterine receptivity in mice. (A) A flow chart overview of this study. (B) The pre‐receptive and the receptive uterus from mice were collected on gestational days (GD) 3 and 4 respectively. (C) The morphology of embryos recovered from the oviduct on GD3 and from the uterus on GD4. (D) Haematoxylin/eosin staining of mouse uterus from GD3 and GD3 showing all the three layers, endometrium, myometrium and perimetrium. (E) Single‐cell RNA‐seq data pre‐processing and quality control. Cells with detected genes fewer than 200 or more than 6000 were removed. Only cells with total mitochondrial gene expression below 25% were kept. (F). Scatter plots showing the correlation between single‐cell RNA‐seq and bulk RNA‐seq. For single‐cell RNA‐seq data, gene expression levels were averaged and normalized as transcripts per million (TPM). For bulk RNA‐seq data, gene expression levels were measured as transcripts per kilobase million (TPM)

To create a cell‐type resolved map of uterine receptivity in mice, we performed single‐cell RNA‐seq analysis (Figure 1A). Pre‐receptive uterus and receptive uterus were collected from gestational days (GD) 3 and 4 respectively (Figure 1B). Success of breeding and early embryo development was confirmed by recovering morula from the oviduct on GD3 and blastocysts from the uterus on GD4 (Figure 1C). The whole uterus, which is consist of endometrium, myometrium and perimetrium, was used for single‐cell suspension preparation (Figure 1D). Single‐cell RNA‐seq data were generated by using the 10x Genomics platform. After quality control, a total of 9337 cells (4108 cells for GD3 uterus and 5229 cells for GD4 uterus) were obtained (Figure 1E). In order to validate this single‐cell RNA‐seq data set, we also generated a bulk RNA‐seq data set using the same samples. The cell‐averaged single‐cell RNA‐seq data were highly accordant with the conventional bulk RNA‐seq data (*r *= 0.8062 for GD3 and *r *= 0.7988 for GD4), indicative of high quality of our single‐cell RNA‐seq data (Figure 1F).

**FIGURE 2:**
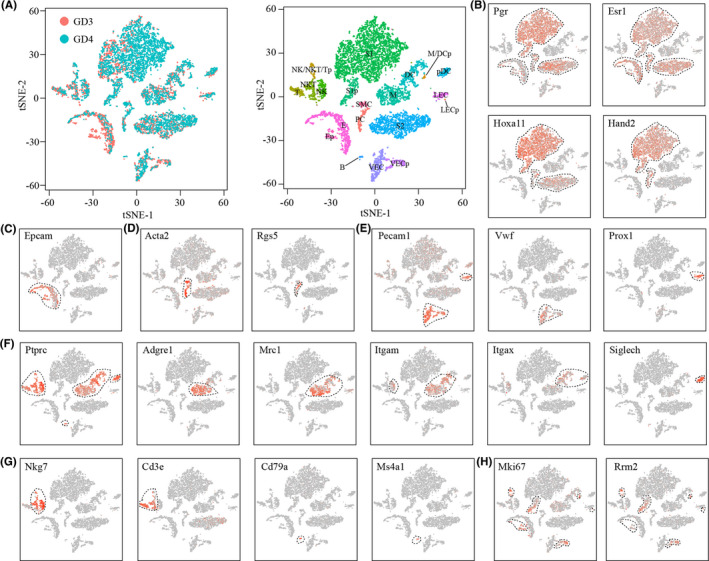
Identification of different cell types in mouse uterus by using canonical gene markers. (A) TSNE visualization of cell clusters in mouse uterus by integrating GD3 and GD4 data. Single cells were grouped by cellular origin (left) and cell clusters (right). E, epithelial cells; Ep, proliferating epithelial cells; S1, superficial stromal cells; S2, deep stromal cells; S1p, proliferating superficial stromal cells; SMC, smooth muscle cells; PC, pericytes; VEC, vascular endothelial cells; VECp, proliferating vascular endothelial cells; LEC, lymphatic endothelial cells; VECp, proliferating lymphatic endothelial cells; M, macrophages; DC, dendritic cells; pDC, plasmacytoid dendritic cells; M/DCp, proliferating mixed macrophages and dendritic cells; NK, natural killer cells; NKT, natural killer T cells; T, T cells; NK/NKT/Tp, proliferating mixed natural killer cells, natural killer T cells and T cells; B, B cells. (B‐H) TSNE plots showing the expression pattern of canonical marker genes for stromal cells (B), epithelial cells (C), smooth muscle cells (D), endothelial cells (E), antigen‐presenting cells (F), lymphocytes (G) and proliferating cells (H). Dashed lines give the boundaries of the specific cell clusters

Unsupervised clustering analysis revealed 19 distinct cell clusters for all cells from GD3 and GD4 combined (Figure 2A). Major cell types were defined using the expression of known cell‐type‐specific genes, with stromal cells expressing Hoxa11,^11^ epithelial cells expressing Epcam and Krt19,^28^ smooth muscle cells expressing Acta2,^29^ pericytes expressing Rgs5,^30^ endothelial cells expressing Pecam1^31^ and immune cells expressing Ptprc.^32^ We found 3 stromal cell clusters, S1, S2 and S1p. Cells in S1 but not in S2 expressed high levels of Hand2 (Figure 2B), implying that S1 was superficial stromal cells and S2 was deep stromal cells.^33^ S1p was linked to S1 and expressed high level of Mki67, suggesting that S1p was a subset of proliferating S1 cells. There were 2 epithelial cell clusters, E and Ep (Figure 2C). Ep was a subset of proliferating E cells with high expression of Mki67. Only one smooth muscle cell cluster (SMC) and one pericyte cluster (PC) were identified (Figure 2D). Endothelial cells have 4 clusters (Figure 2E): VEC and its proliferating subset VECp were vascular endothelial cells expressing Vwf,^31^ while LEC and its proliferating subset LECp were lymphatic endothelial cells expressing Prox1.^31^ The 8 immune cell clusters are macrophages (M, Ptprc^+^Adgre1^+^ ^34^), dendritic cells (DC, Ptprc^+^Adgre1^−^Itgax^+^ ^34^), plasmacytoid dendritic cells (pDC, Ptprc^+^Siglech^+^ ^35^), natural killer cells (NK, Ptprc^+^Nkg7^+^Cd3e^−^ ^36^), natural killer T cells (NKT, Ptprc^+^Nkg7^+^Cd3e^+^ ^36^), T cells (T, Ptprc^+^Nkg7^−^Cd3e^+^ ^36^), B cells (B, Ptprc^+^Cd79a^+^ ^36^), a proliferating subset of mixed macrophages and dendritic cells (M/DCp, Ptprc^+^Adgre1^+^Mki67^+^ or Ptprc^+^Itgax^+^Mki67^+^), and a proliferating subset of mixed natural killer cells, natural killer T cells and T cells (NK/NKT/Tp, Ptprc^+^Nkg7^+^Mki67^+^ or Ptprc^+^Cd3e^+^Mki67^+^) (Figure 2F–H).

We then re‐analysed a single‐cell RNA‐seq data set for non‐pregnant adult mouse uterus from the mouse cell atlas project.^37^ Using the same computational pipeline, we identify LE, GE, S1, S2, SMC, PC, VEC, LEC, M, DC, pDC and NK. However, NKT, T or B were not found (Fig. S1). This analysis indicated that the markers used for receptive uterus can also be used for non‐pregnant adult mouse uterus.

We next aimed to discover novel markers for each cell type. We selected genes that were expressed significantly higher in the cell type of interest than the other cell types by Wilcoxon rank sum test. A heat map depicting the top 10 marker genes for each cell type is shown in Fig. S2A. A complete list of marker genes was presented in Table S1. We picked up a single‐marker gene for all cell types except DC and SMC (Fig. S2B). We found that these novel marker genes were as good as, if not better than, canonical marker genes.

Finally, we searched for endometrial epithelial stem/progenitor cells and endometrial mesenchymal stem cells. The marker genes for endometrial epithelial stem/progenitor cells were Cd44,^38^ Lrg5,^39^ Fut4^40^ and Aldh1a1,^41^ while the marker genes for endometrial mesenchymal stem cells were Susd2^42^ and Ncam.^43^ We found that there was little overlap in expression pattern between these marker genes. We also found that these potential stem/progenitor cells were not clustered but rather scattered among other cells in the TSNE plots (Fig. S3). Further validations are needed to confirm our findings.

### 3.2 *Cell* *type*‐*specific transcriptional changes associated with uterine receptivity*

**FIGURE 3:**
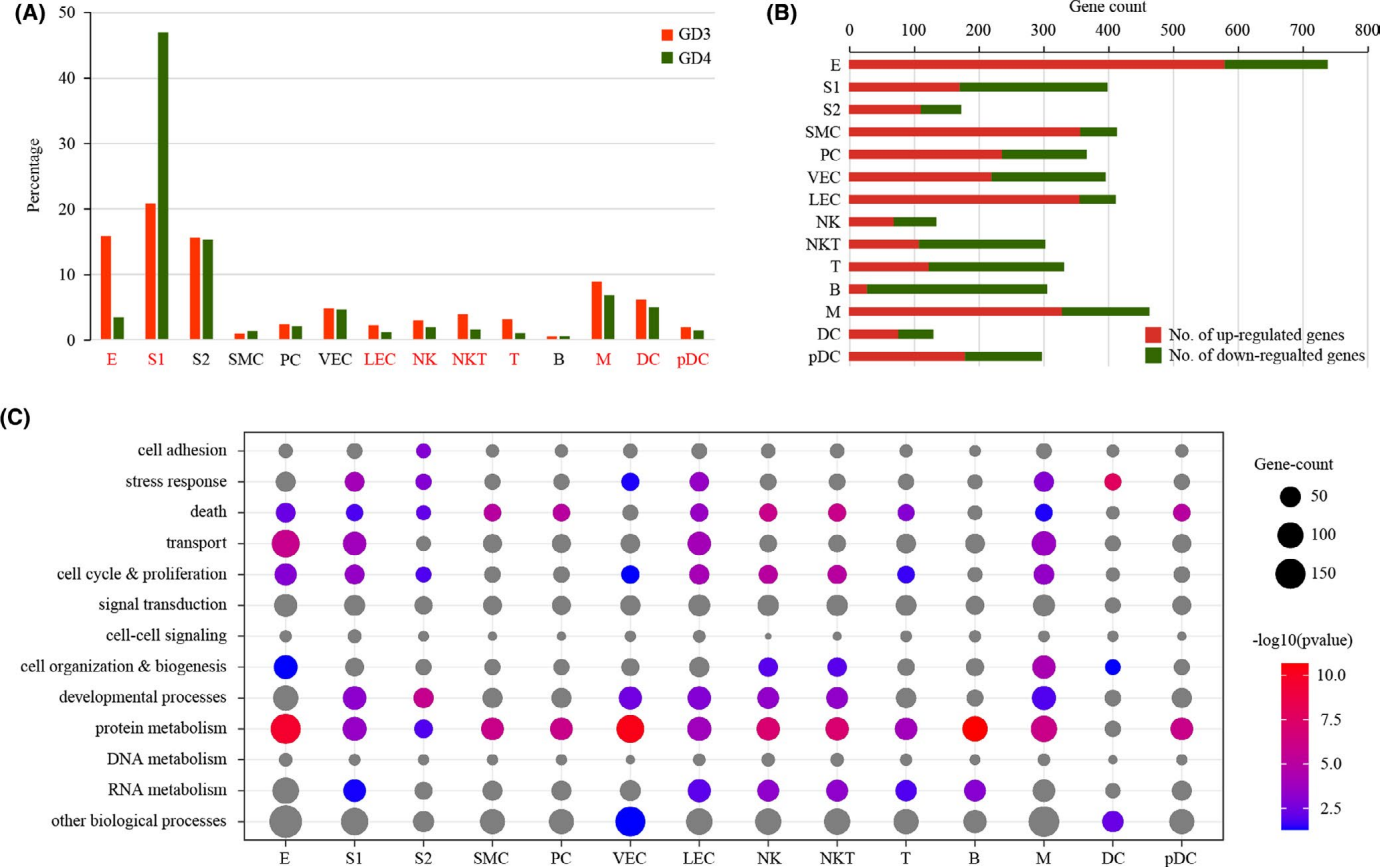
Cell population shifts and gene expression changes in receptive uterus compared to pre‐receptive uterus. (A) Bar plot showing the cell population change of 14 major cell types in mouse uterus on GD4 compared to GD3. Cell types with *p* < 0.05 by chi‐square test were labelled in red. (B) The distribution of differentially expressed genes in each cell type (logFC >0.25 and *p* < 0.05). (C) Gene ontology enrichment analysis of differentially expressed genes. Significant hits (*p* < 0.05) were shown as colour circles, while non‐significant ones were displayed in grey

We investigated the abundance of each cell type (Figure 3A). The proportions of S2, SMC and PC were unchanged in GD4 uterus compared to GD3 uterus. The proportion of S1 was significantly increased, whereas the proportions of E and immune cells (M, DC, pDC, NK, NKT and T) were significantly decreased. Notably, although the proportion of VEC was unchanged, the proportion of LEC was significantly reduced in GD4 uterus compared to GD3 uterus.

We investigated the breadth of transcriptional changes in each cell type by performing differential gene expression analysis. Using a logFC cut‐off of 0.25 and a p value cut‐off of 0.05, we identified 739, 399, 173, 414, 367, 396, 412, 464, 130, 298, 135, 303, 332 and 306 differentially expressed genes for E, S1, S2, SMC, PC, VEC, LEC, M, DC, pDC, NK, NKT, T and B respectively (Figure 3B and Table S2). We then explored the biological implications of differentially expressed genes using gene ontology (GO) analysis. A complete list of enriched GO terms was provided in Figure 3C. These data indicated that each cell type invokes distinct biological processes in order to participate in the establishment of uterine receptivity.

### 3.3 *Dissecting* *luminal and glandular epithelium from single*‐*cell RNA*‐*seq data*

**FIGURE 4:**
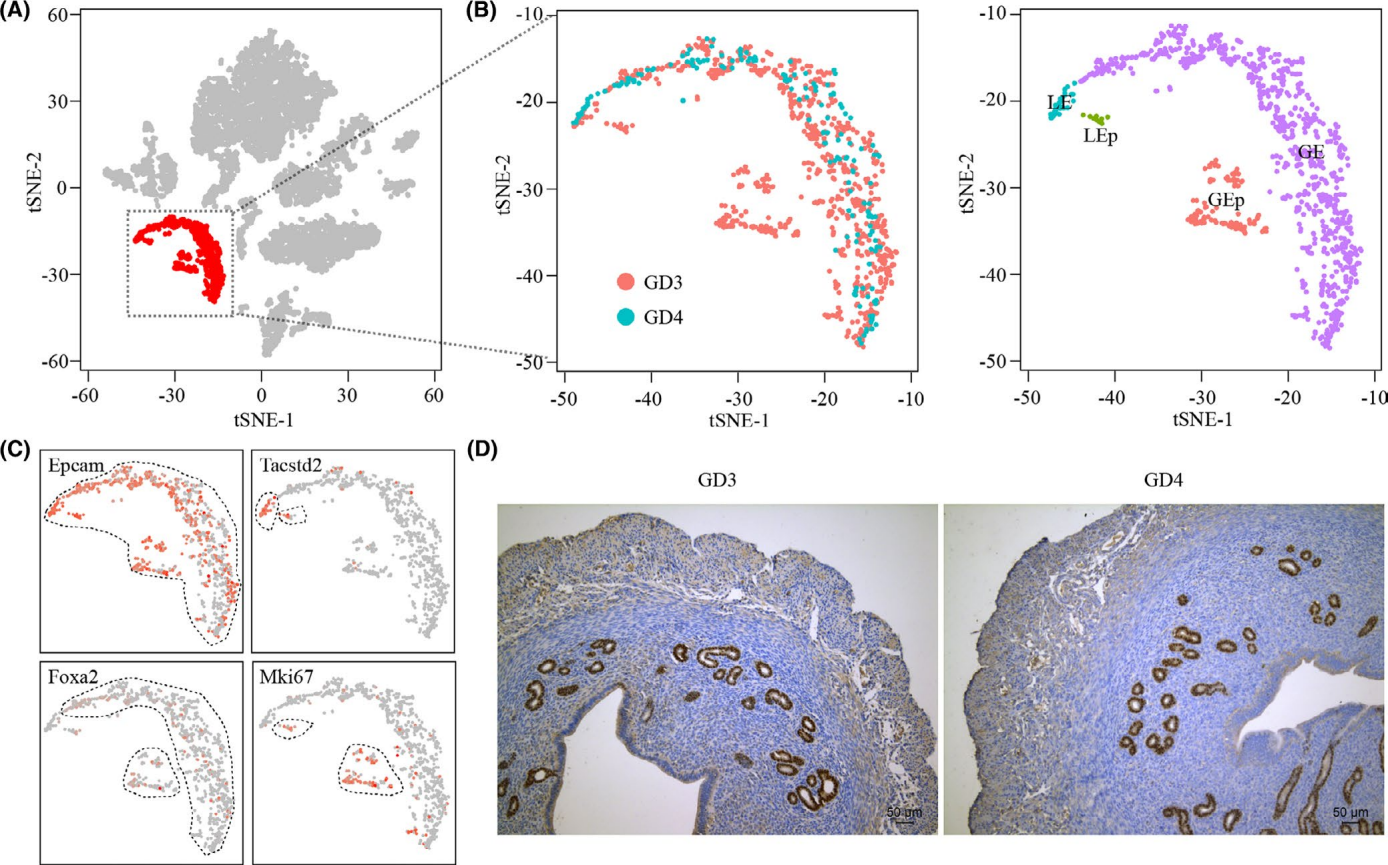
Dividing epithelial cells into sub‐clusters. (A) Selection of epithelial cells from uterine cells. (B) Visualizing sub‐clusters of epithelial cells by TSNE plot. LE, luminal epithelial cells; LEp, proliferating luminal epithelial cells; GE, glandular epithelial cells; GEp, proliferating glandular epithelial cells. (C) The expression pattern of marker genes for sub‐clusters of epithelial cell. (D) Immunohistochemical analysis of Foxa2 expression. Bar = 50 μm

We focussed on uterine epithelium (Figure 4A), because it is the first maternal contact for embryo implantation.^44^ Using Tacstd2 as luminal epithelium (LE) marker and Foxa2 as glandular epithelium (LE) marker,^45^ we divided uterine epithelium into 4 cell clusters: LE, proliferating LE (LEp), GE and proliferating GE (GEp) (Figure 4B and Figure 4C). LEp and GEp were found only in GD3 uterus. We observed that Foxa2 was only expressed in a small portion of GE cells in our single‐cell RNA‐seq data. By using Immunohistochemistry, we found that Foxa2 was actually expressed in almost all GE cells (Figure 4D). This was likely a ‘dropout’ phenomenon commonly seen in single‐cell RNA‐seq experiments: some mRNA molecules might be lost due to their tiny initial amounts in the library preparation step, leading to false zero values.^46^

**FIGURE 5:**
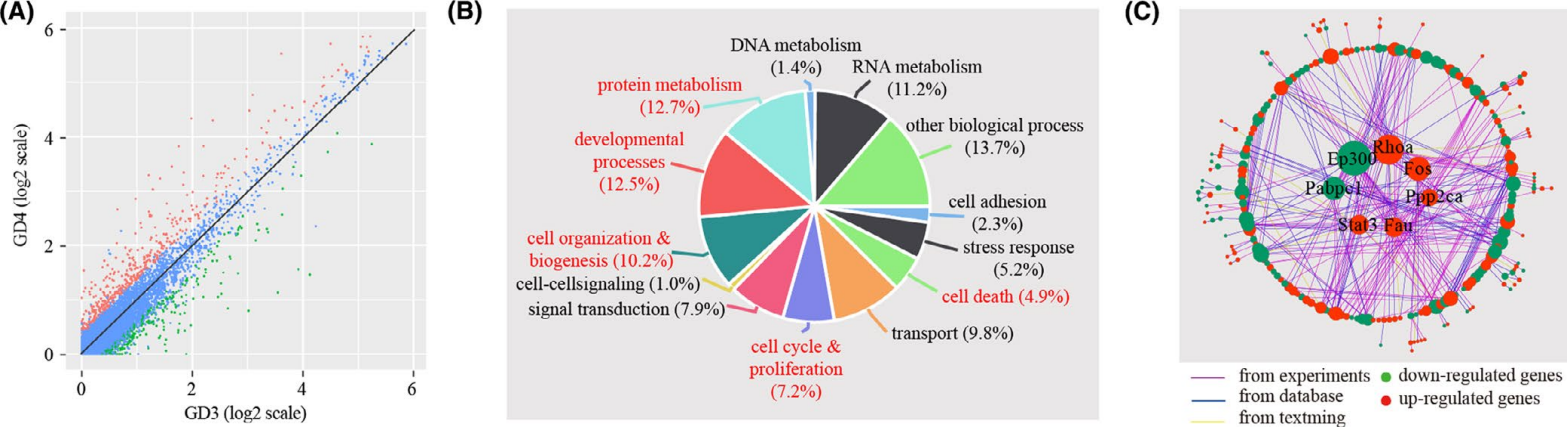
Identification of differentially expressed genes in LE cells. (A) Scatter plot for the comparison of gene expression levels in LE cells between GD3 and GD4. The threshold values for differentially expressed genes were logFC >0.25 and *p* < 0.05. Down‐regulated genes, up‐regulated gene and non‐changed genes were shown in green, red and blue respectively. (B) Gene ontology (GO) enrichment analysis of differentially expressed genes. Differentially expressed genes were grouped based on MGI GOslim terms under the biological process categories. Significantly enriched GO terms (*p* < 0.01) were coloured in red. (C) Gene network underlying differentially expressed genes. Up‐regulated genes were coloured in red, and down‐regulated genes were coloured in green. Hub genes, which are defined as genes with degree values exceeding the mean plus two standard deviations, were displayed in the centre of the network

In LE, we found that a total of 749 genes were differentially expressed, of which 296 genes were down‐regulated and 453 genes were up‐regulated on GD4 in comparison to GD3 (Figure 5A and Table S3). Functional clustering analysis categorized the differentially expressed genes into 12 biological processes (Figure 5B): cell adhesion (2.3%), stress response (5.2%), cell death (4.9%), transport (9.8%), cell cycle & proliferation (7.2%), signal transduction (7.9%), cell‐cell signalling (1.0%), cell organization & biogenesis (10.2%), developmental processes (12.5%), protein metabolism (12.7%), DNA metabolism (1.4%), RNA metabolism (11.2%) and other biological processes (13.7%). Based on the enrichment test, 5 out of these GO terms, namely cell death (*p *= 0.00018), cell cycle & proliferation (*p *= 0.000015), cell organization & biogenesis (*p *= 0.0000049), developmental processes (*p *= 0.00011) and protein metabolism (*p *= 0.00028), were significantly enriched among differentially expressed genes (*p *< 0.01). Additionally, we reconstructed a network for differentially expressed genes by using the STRING online tool. This network had 242 genes with 518 edges (Figure 5C). Within this network, some nodes, known as hub genes, are highly connected compared to others. Using a defined cut‐off value, we identified 7 hub genes: Pabpc1, Ep300, Rhoa, Fos, Ppp2ca, Fau and Stat3.

**FIGURE 6:**
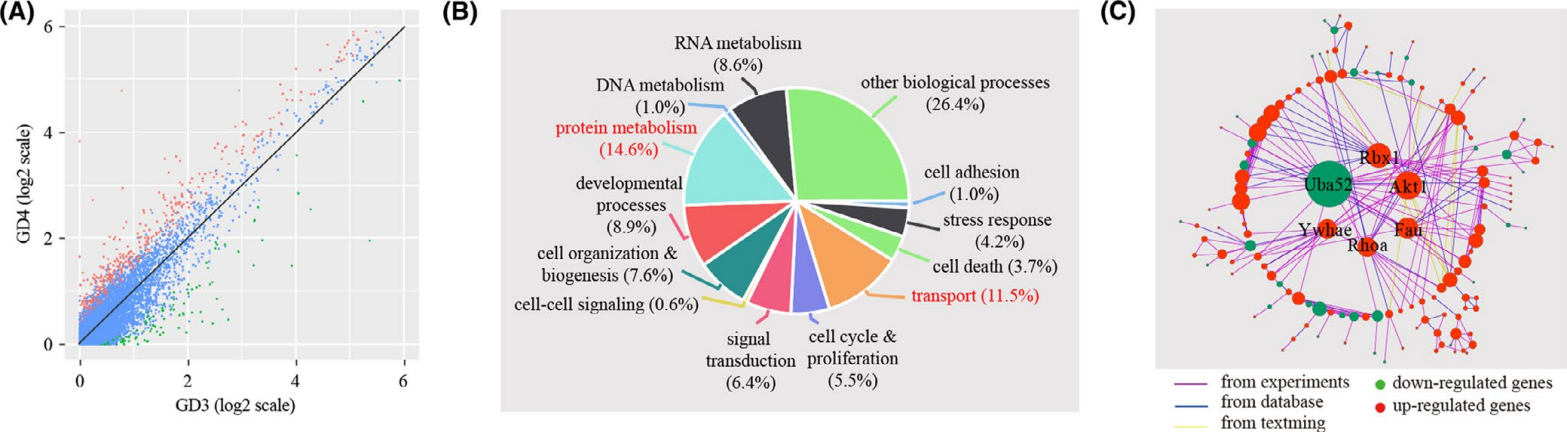
Identification of differentially expressed genes in GE cells. (A) Scatter plot for the comparison of gene expression levels in GE cells between GD3 and GD4. Non‐changed genes were marked in blue, while differently expressed genes (logFC >0.25 and *p* < 0.05) were denoted in red or green. (B) GO enrichment analysis of differentially expressed genes. Significantly enriched GO terms (*p* < 0.01) were coloured in red. (C) Gene network for differentially expressed genes. Hub genes were displayed in the centre of the network

In GE, we identified a total of 595 differentially expressed genes, of which 149 genes were down‐regulated and 446 genes were up‐regulated on GD4 compared to GD3 (Figure 6A and Table S4). According to gene ontology (GO), differentially expressed genes can be categorized into 12 biological processes (Figure 6B): cell adhesion (1.0%), stress response (4.2%), cell death (3.7%), transport (11.5%), cell cycle & proliferation (5.5%), signal transduction (6.4%), cell‐cell signalling (0.6%), cell organization & biogenesis (7.6%), developmental processes (8.9%), protein metabolism (14.6%), DNA metabolism (1.0%), RNA metabolism (8.6%) and other biological processes (26.4%). Based on hypergeometric test, transport (*p *= 0.00038) and protein metabolism (*p *= 0.00000015) were significantly enriched. We constructed a gene network for differently expressed genes, consisting of 139 nodes connected via 252 edges (Figure 6C). We found that Uba52, Rbx1, Akt1, Fau, Rhoa and Ywhae were hub genes of this network.

### 3.4 *Validating* *single*‐*cell RNA*‐*seq data by laser capture microdissection*‐*coupled microarray data*

**FIGURE 7:**
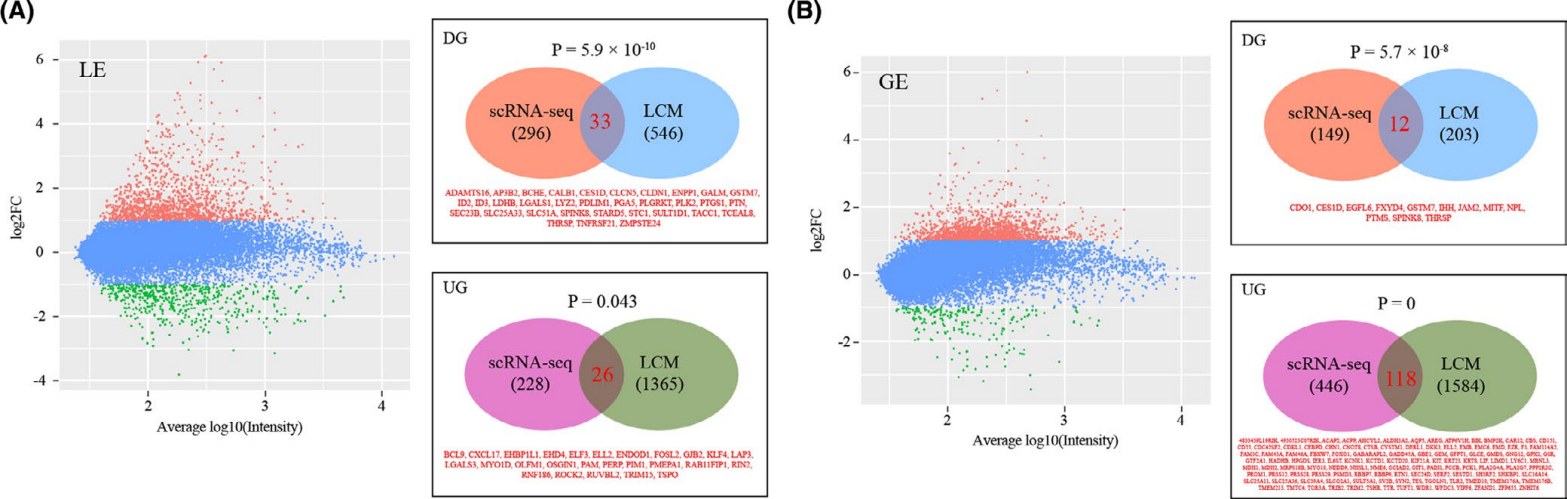
Validating single‐cell RNA‐seq data by laser capture microdissection (LCM)‐coupled microarray data. (A) A comparison of differential expressed genes in LE. Left: MA plot showing differential expressed genes in LE in the LCM‐coupled microarray data. Fold change >2 was used to select differently expressed genes. Down‐regulated genes, up‐regulated gene and non‐changed genes were shown in green, red and blue respectively. Right: Venn diagram showing the overlap of differentially expressed genes between our single‐cell RNA‐seq data and LCM‐coupled microarray data. (B) A comparison of differential expressed genes in GE. Left: MA plot showing differential expressed genes in LE in LCM‐coupled microarray data. Right: Venn diagram showing the overlap of differentially expressed genes between our single‐cell RNA‐seq data and LCM data. DG, down‐regulated genes; UG, up‐regulated genes. P values were calculated using the hypergeometric test, and the background parameter N was set to 15963

Previously, microarray analysis was conducted on LE and GE isolated by laser capture microdissection (LCM) from uterus at 1600 h on GD3 and GD4 of pseudo‐pregnancy.^47^ In order to further validate our single‐cell RNA‐seq data set, a comparison analysis was performed. LCM microarray data were downloaded from GEO database under the accession number GSE48239. Fold change >2 was used to select differently expressed genes. In LE, we identified 33 overlap down‐regulated genes and 26 overlap up‐regulated genes respectively (Figure 7A). In GE, we found 12 overlap down‐regulated genes and 118 overlap up‐regulated genes respectively (Figure 7B). Notably, *p* < 0.05 was reached for all comparison, providing validity of our single‐cell RNA‐seq data.

### 3.5 *Inferring* *cell*‐*cell communication between blastocysts and the receptive uterus*

**FIGURE 8:**
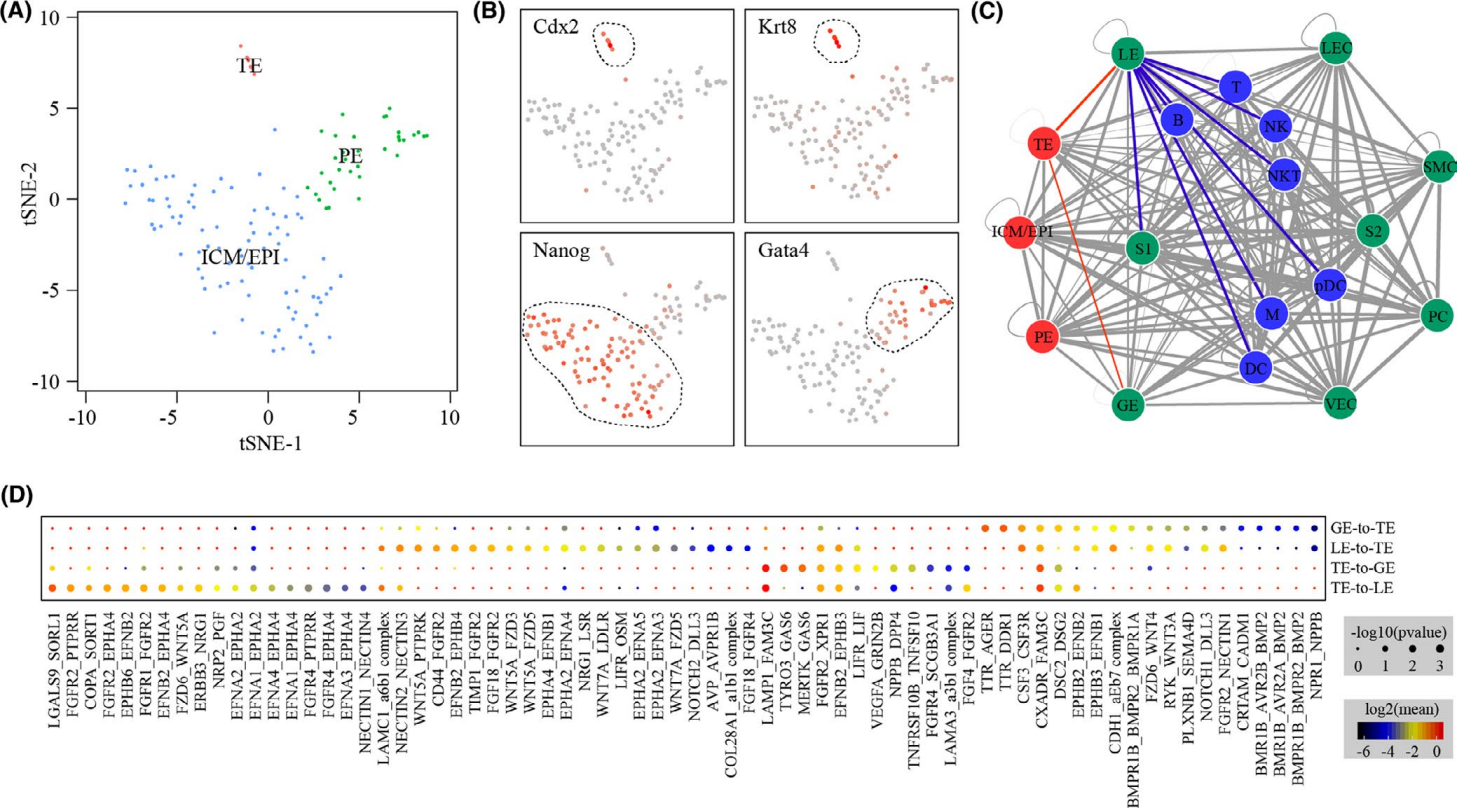
Ligand‐receptor interactions between the blastocyst and the uterus on GD4. (A) TSNE clustering of single cells from mouse E3.5 blastocysts which were recovered from GD4 uterus. TE, trophectoderm; ICM/EPI, inner cell mass/epiblast; PE, primitive endoderm. (B) TSNE map showing the expression pattern of well‐known marker genes. (C) Network plot showing the ligand‐receptor interaction events between blastocysts and the uterus on GD4. Cell‐cell communication is indicated by the connected line. The thickness of the lines is correlated with the total number of ligand‐receptor interaction events. The interactions that are likely associated with the establishment of uterine receptivity were coloured in red. The red node indicates cells from blastocysts. The green nodes and the blue nodes are non‐immune and immune cells from the uterus respectively. Abbreviations for cell types are listed in Figure 2. (D) Dot plot showing selected ligand‐receptor interactions underlying TE‐LE and TE‐GE crosstalk. P values are indicated by circle size, and means of the average expression level of interacting molecule are indicated by colour

On GD4, the embryo, which is denoted as E3.5 blastocysts, enters the uterus from the oviduct. We re‐analysed a published 10x single‐cell RNA‐seq data set on mouse E3.5 blastocysts^26^ (Figure 8A). By using canonical marker genes, 3 major cell types were identified: trophectoderm (TE), inner cell mass/epiblast (ICM/EPI) and primitive endoderm (PE) (Figure 8B). To investigate the crosstalk between E3.5 blastocysts and GD4 uterus, we used CellPhoneDB to predict the ligand‐receptor interactions between distinct cell types (Figure 8C). Of special interest, 70 TE‐LE/TE‐GE ligand‐receptor interaction pairs were identified (Figure 8D). Considering spatial relationships between cell types, these ligand‐receptor interactions might play a crucial role in the establishment of uterine receptivity.

### 3.6 *Comparison* *of uterine receptivity between mice and humans at single*‐*cell resolution*

**FIGURE 9:**
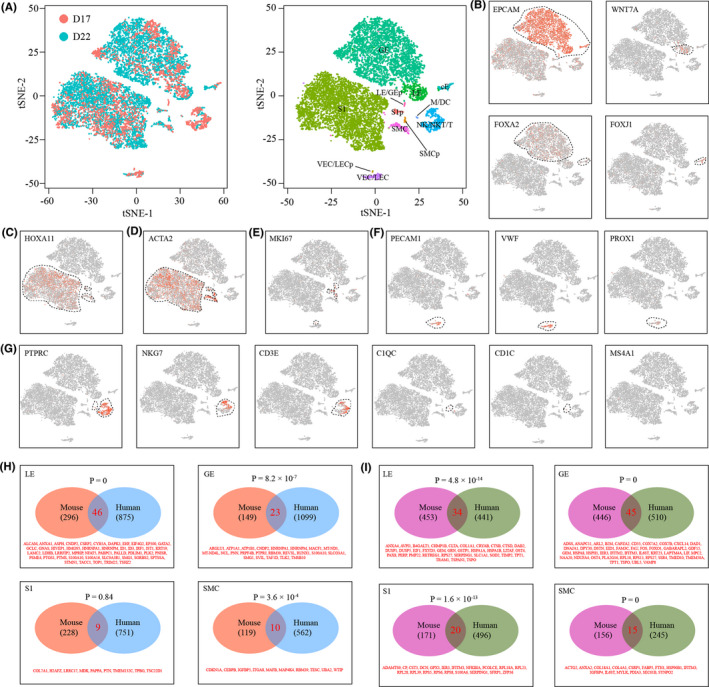
Comparison of uterine receptivity between mice and humans at single‐cell resolution. (A) TSNE visualization of single‐cell RNA‐seq data collected from the pre‐receptive phase (day 17 of the menstrual cycle, D17) and the receptive phase (day 22 of the menstrual cycle, D17) of human endometrium. Single cells were coloured by tissue source (left) and cell clusters (right). LE, epithelial cells; GE, glandular epithelial cells; cE, ciliated epithelial cells (cE); LE/GEp, proliferating mixed luminal and glandular epithelial cells; S1, superficial stromal cells, S1p, proliferating stromal cells; SMC, smooth muscle cells; SMCp, proliferating smooth muscle cells; M/DC, mixed macrophages and dendritic cells; NK/NKT/T, mixed NK, NKT and T cells; VEC/LEC, mixed vascular and lymphatic endothelial cell; VEC/LECp, proliferating mixed vascular and lymphatic endothelial cells. (B‐G) TSNE plots showing the expression pattern of canonical marker genes for epithelial cells (B), stromal cells (C), smooth muscle cells (D), proliferating cells (E), endothelial cells (F) and immune cells (G). (H‐I) Venn diagram showing the overlap of differentially expressed genes between mice and humans in 4 major cell types. Down‐regulated genes (H) and up‐regulated genes (I) were compared seperately. P values were calculated using the hypergeometric test. The background N was set to 20366, and this parameter was estimated by averaging single‐cell RNA‐seq data to produce pseudo‐bulk RNA‐seq data

Finally, we compared our single‐cell RNA‐seq data with the a recently published single‐cell RNA‐seq data set which was generated from the pre‐receptive phase (day 17 of the menstrual cycle, D17) and the receptive phase (day 22 of the menstrual cycle, D22) of human endometrium by using the 10x Genomic method.^48^ The same computational pipeline was employed to re‐analyse this data set (Figure 9A). We identified a total of 12 cell clusters, namely luminal epithelial cells (LE), glandular epithelial cells (GE), ciliated epithelial cells (cE), proliferating mixed luminal and glandular epithelial cells (LE/GEp), stromal cells (S1), proliferating stromal cells (S1p), smooth muscle cells (SMC), proliferating smooth muscle cells (SMCp), mixed macrophages and dendritic cells (M/DC), mixed NK, NKT and T cells (NK/NKT/T), mixed vascular and lymphatic endothelial cells (VEC/LEC) and proliferating mixed vascular and lymphatic endothelial cells (VEC/LECp) (Figure 9B–G). We then attempted to compare differentially expressed genes associated with uterine receptivity between mice and humans in 4 stringently comparable cell types, namely LE, GE, S1 and SMC. For down‐regulated genes, a significant overlap was found in LE, GE and SMC, but not in S1 (Figure 9H). For up‐regulated genes, a significant overlap was found in all these 4 cell types (Figure 9I). These data highlighted the similarity in uterine receptivity between mice and humans.

## 4 DISCUSSION

The mouse is widely used as an animal model for investigating human uterine receptivity. In this study, by profiling the single‐cell transcriptome for 9337 cells using the 10x Genomics approach, we revealed 19 distinct cell clusters, including 3 stromal cell clusters, 2 epithelial cell clusters, 1 smooth muscle cell cluster, 4 endothelial cell clusters and 8 immune cell clusters. To the best of our knowledge, the present study is the first to highlight the transcriptome landscape of mouse pre‐receptive and receptive uterus at single‐cell resolution.

Cell dissociation is a prerequisite for single‐cell RNA‐seq studies. In a previous study,^30^ mouse uterus from post‐natal day 12 was digested with 10 mg/ml *Bacillus licheniformis* enzyme. In another study,^48^ human cycling endometrium biopsy was digested with collagenase A1 and then TrypLE Select enzyme in a two‐step procedure. In this study, we used 2 mg/ml Collagenase II and 10 mg/ml Dispase II for single‐cell suspension preparation. Collagenase II is a crude collagenase preparation with weak trypsin‐like activity. Thus, trypsin was not used in our method. As a result, the cell viability was >80% and the percentage of cell clumps was <10%, indicative of high efficiency of our method. However, we would like to note that whatever digesting enzymes were used, they might cause transcriptional disturbances of varying degree, leading to artefacts.^49^ For example, we found that the expression of Il1b was zero in bulk RNA‐seq data obtained from snap‐frozen pre‐receptive and receptive uterus, whereas high expression of Il1b was observed when we averaged single‐cell RNA data to produce pseudo‐bulk RNA‐seq data. By examining our single‐cell RNA‐seq data, we found that Il1b was specially expressed in macrophages. We considered the high expression of Il1b in macrophages as an artefact caused by the cell dissociation procedure. To what extend different cell dissociation procedure disturbed cellular transcriptome awaits further investigations.

Based on our single‐cell RNA‐seq data, the cell‐type composition for pre‐receptive uterus on GD3 is 40.6% stromal cells, 17.6% epithelial cells, 3.6% smooth muscle cells, 7.8% endothelial cells and 30.4% immune cells, while the cell‐type composition for receptive uterus on GD4 is 67.0% stromal cells, 3.7% epithelial cells, 3.7% smooth muscle cells, 6.1% endothelial cells and 19.5% immune cells. Of note, the estimated percentages for each cell type may be distorted from their actual proportions in the mouse uterus, as the recovery rate for each cell type might vary during the cell dissociation procedure. Nevertheless, because pre‐receptive and receptive uterus samples were processed in parallel with the same protocol, we expected that the changes in cell‐type composition ratio might reflect a real biological effect. We found that although the proportion of deep stromal cells remained unchanged, the proportion of superficial stromal cells was significantly increased, which is line with the observation that there were more proliferating superficial stromal cells in GD4 uterus compared to GD3 uterus. On the other hand, we found that the proportion of epithelial cells was significantly reduced in GD4 uterus compared to GD3 uterus. Since both proliferation and apoptosis of epithelial cells cease on GD4,^44^ it was likely a result of the large amount of new superficial stromal cells on GD4 which might dilute the proportion of epithelial cells. Additionally, we found the proportion of immune cells was significantly reduced. Considering that proliferating immune cells were increasing on GD4, this was likely also a dilution effect.

We investigated the breadth of transcriptional changes for each cell type by performing differential gene expression analysis. As expected, the epithelial cells have the largest number of differentially expressed genes. We further divided epithelial cells into luminal epithelium (LE) and the glandular epithelium (GE) by using known gene markers. We identified 749 and 595 differentially expressed genes for LE and GE respectively. Gene ontology analysis revealed that cell death, cell cycle & proliferation, cell organization & biogenesis, developmental processes and protein metabolism were significantly enriched among differentially expressed genes in LE, while transport and protein metabolism were significantly enriched among differentially expressed genes in GE. Gene prioritization was performed by selecting hub genes from gene network. We identified 7 hub genes (Pabpc1, Ep300, Rhoa, Fos, Ppp2ca, Fau and Stat3) for LE and 6 hub genes (Uba52, Rbx1, Akt1, Fau, Rhoa and Ywhae) for GE. Fau and Rhoa were mutual hub genes for LE and GE. Due to their key positions in the network, hub genes are supposed to be more important than the others. Thus, these hub genes deserve further investigation.

In order to further validate our single‐cell RNA‐seq data set, we re‐analysed a microarray data set conducted on LE and GE isolated by LCM from uterus at 1600 h on GD3 and GD4 of pseudo‐pregnancy.^47^ Fold change >2 was used to select differently expressed genes in the LCM microarray data. By using statistical test, we found significant overlaps in differentially expressed genes between single‐cell RNA‐seq data and LCM microarray data for both LE and GE. These findings provided validity of our single‐cell RNA‐seq data.

In this study, the whole uterus was obtained on GD3 and GD4 respectively. Success of breeding and early embryo development was confirmed by recovering morula from the oviduct on GD3 and blastocysts from the uterus on GD4. In both cases, the un‐flushed uterus was used for sample collection. Therefore, our samples might contain embryos. An embryo is typically of <100 cells. We usually obtained >0.5 million cells per sample in the single‐cell dissociation procedure. Approximately 5000 cells were sequenced per sample by the 10x platform. By calculating the probability, we found that only 1 cell per embryo could be captured in our single‐cell RNA‐seq data. This was in line with our findings that there were no embryo‐derived cell clusters in our single‐cell RNA‐seq data based on canonical gene markers. Previously, the E3.5 blastocysts flushed from GD4 uterus have been subjected to single‐cell RNA‐seq.^26^ Through data integration, we inferred cell‐cell communication between E3.5 blastocysts and receptive GD4 uterus by their expression of ligand‐receptor pairs. We are particularly interested in the interactions between TE from the blastocyst and LE from the uterus, because physical contact between these two cell types represents the initial stage of embryo implantation. Notably, LE cells expressed Wnt proteins (Wnt5a, Wnt7a and Wnt4), while the corresponding receptors (Fzd3, Fzd5 and Fzd6) were expressed in TE, highlighting the importance of Wnt signalling pathway. Additionally, we found that ephrin signalling pathway and notch signalling pathway might also play a significant role in LE‐TE interaction. We were also interested in the interactions between TE and GE. It is well known that Lif secreted from GE of the uterus is required for the establishment of uterine receptivity.^13^ We found that Lifr was expressed in TE. Lif null blastocysts were able to implant and develop to term if transferred to wild‐type pseudo‐pregnant recipients.^13^ Lifr null blastocysts were also able to implant, but normal placentation was disrupted leading to poor intrauterine nutrition.^50^ Thus, it seems that the GE/TE Lif/Lifr axis is not a requirement for embryo implantation. Additionally, we found that Bmp2 secreted by GE might communicate with TE via its receptors.

Due to spatial relationships, uterine cell types other than LE and GE were unable to directly communicate with TE during the peri‐implantation period. Previous studies have shown that uterine epithelial‐stromal crosstalk is crucial for embryo implantation.^51^ We suspected that superficial stromal cells, as well as immune cells, might contribute to uterine receptivity indirectly by influencing LE and GE. Uterine epithelial‐stromal crosstalk revealed by the CellPhoneDB software was provided in Table S5.

In humans, uterine receptivity has a peak on days 20–24 of a regular 28‐day menstrual cycle. In this study, we re‐analysed a published 10x single‐cell RNA‐seq data set on pre‐receptive endometrium (day 17) and receptive endometrium (day 22). By examining cell types, we observed serval differences between mice and humans. Firstly, a cell cluster of ciliated epithelial cells (EPCAM^+^FOXJ1^+^) was unique to the human data set. Whether this discrepancy was due to the technical bias or real species‐specific difference remains to be tested. Secondly, macrophages and dendritic cells could not be clearly separated and were presented in a mixed cell cluster (M/DC) in the human data set. Similarly, there was only a mixed cell cluster for NK, NKT and T cells (NK/NKT/T) and a mixed cell cluster of VEC and LEC (VEC/LEC) in the human data set. Lastly, we found only one cell cluster of stromal cells in the human data set, instead of the 2 cell clusters (S1 and S2) in our mouse data set. The human data set was obtained using endometrial biopsy, whereas our mouse data set was generated form the whole uterus. Therefore, we designated the human stromal cell cluster as an equivalent of superficial stromal cells (S1) in our mouse data set. Nevertheless, by using statistical test, we found significant overlaps in differentially expressed genes between mice and humans for 4 cell types, including LE and GE. For example, Gata2 expression was consistently down‐regulated in LE for mice and humans. In mice, Gata2 is an upstream regulator of Pgr.^52^ Decreased Pgr on the evening of GD4 is required for preparation of uterine receptivity for embryo attachment in mice.^53^, ^54^ The down‐regulation of Pgr in receptive uterus was also observed in humans^55^ and rhesus monkeys.^56^ Therefore, the down‐regulation of Gata2‐Pgr axis is likely a conserved mechanism for the establishment of uterine receptivity. Another example is Lif. We found that Lif was consistently up‐regulated in GE in mice and humans. In mice, Lif transiently increases in in glandular epithelium of mouse on the morning of GD4. LIF null female mice are infertile due to implantation failure.^13^ In humans and rhesus monkeys, LIF is expressed in the endometrial glands during the luteal phase of the menstrual cycle.^57^, ^58^ Intrauterine injection of LIF antibodies reduced embryo implantation rate in rhesus monkeys.^57^ Systemic administration LIFR antagonist PEGLA blocked LIF activity and prevented implantation in cynomolgus monkeys.^59^ Thus, the Lif pathway may play a consensus role in uterine receptivity.

In conclusion, this study provided a comprehensive single‐cell transcriptome atlas for mouse pre‐receptive uterus and receptive uterus. Our data present a valuable resource for deciphering the molecular mechanism underlying uterine receptivity.

## CONFLICT OF INTEREST

The authors declare that there is no conflict of interest that could be perceived as prejudicing the impartiality of the research reported.

## AUTHOR CONTRIBUTIONS

J.‐L.L supervised the study. J.‐L.L designed the experiments. Y.Y. and Q.‐Y.Z performed the experiments. Y.Y. and J.‐L.L analysed the data. Y.Y. and J.‐L.L wrote the paper. All authors read and approved the final paper.

## Supporting information

Fig S1

Fig S2

Fig S3

Table S1

Table S2

Table S3

Table S4

Table S5

## Data Availability

All raw and analysed sequencing data can be found at Gene Expression Omnibus.
