# Supplementary figures and images for: Deciphering mouse uterine receptivity for embryo implantation at single‐cell resolution
Source: Cell Prolif. 2021 Sep 23;54(11):e13128. doi: 10.1111/cpr.13128 (PMC8560620; doi:10.1111/cpr.13128)

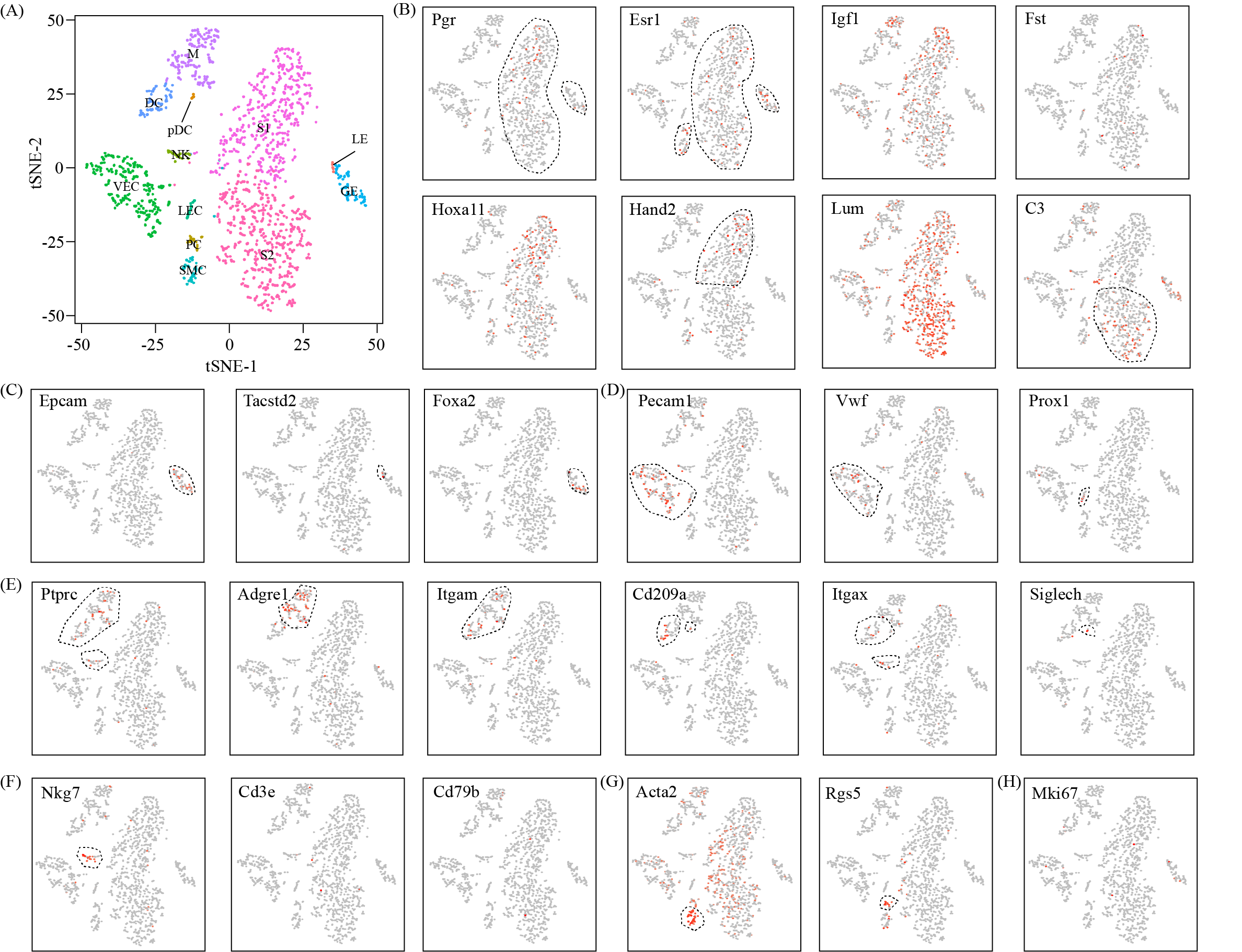

Supplement: Supplementary file 1 — Fig S1 [file CPR-54-e13128-s006.tif]

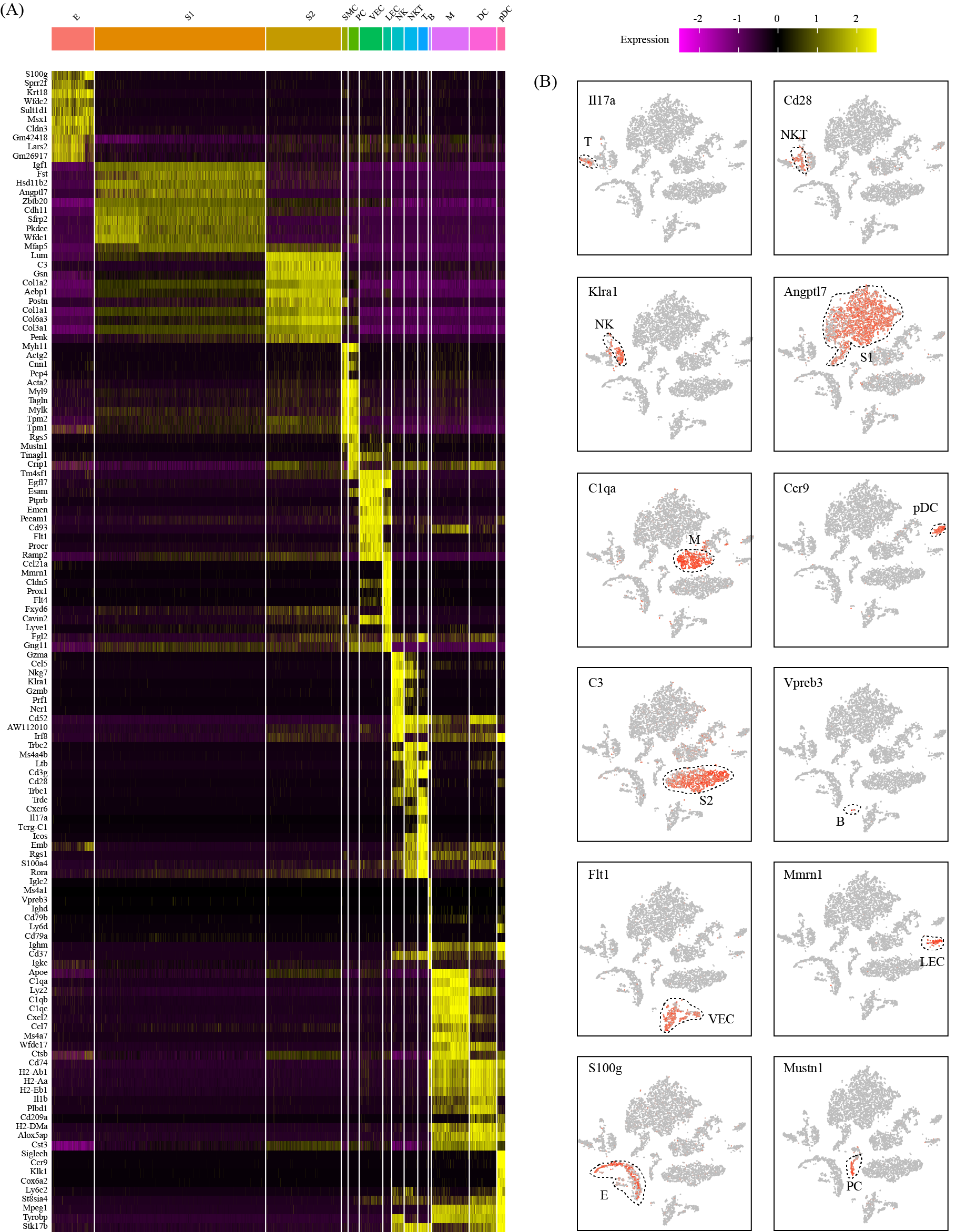

Supplement: Supplementary file 2 — Fig S2 [file CPR-54-e13128-s002.tif]

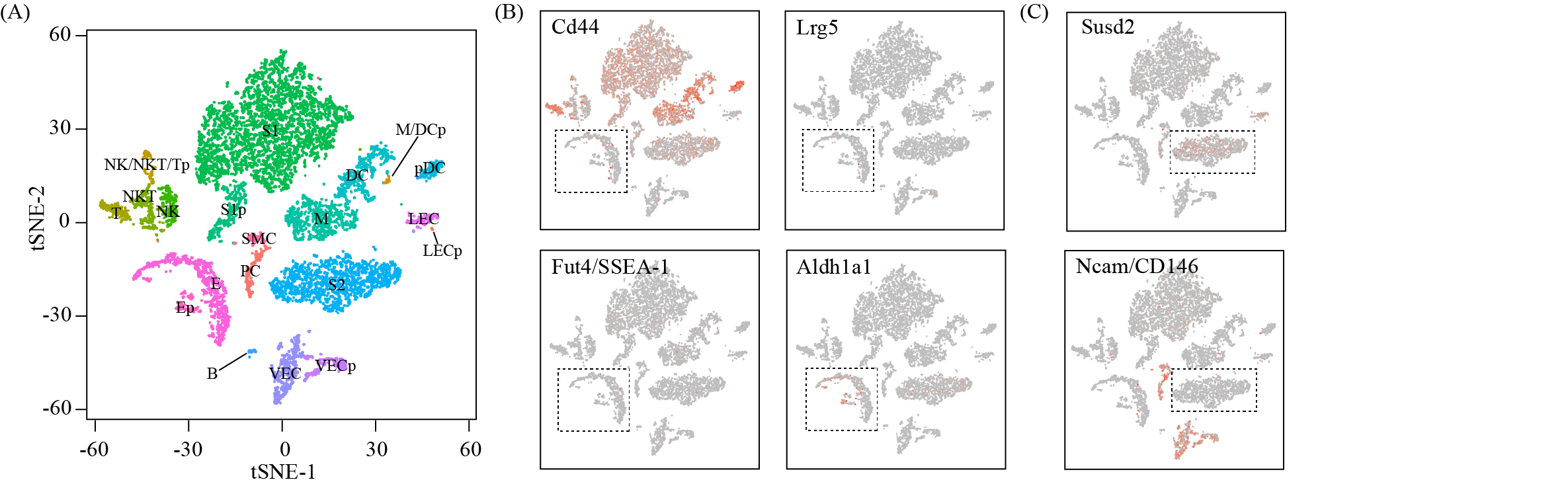

Supplement: Supplementary file 3 — Fig S3 [file CPR-54-e13128-s001.tif]
